# Clove Essential Oil as a Source of Antitumoral Compounds Capable of Crossing the Blood–Brain Barrier: A Focus on the Effects of β-Caryophyllene and Eugenol in a Glioblastoma Cell Line

**DOI:** 10.3390/ijms26010238

**Published:** 2024-12-30

**Authors:** Renato Spigarelli, Enzo Spisni, Mariana Magalhães, Célia Cabral, Ana Cristina Gonçalves, Ilaria Maria Saracino, Giada Botti, Alessandro Dalpiaz, Sarah Beggiato, Maria Chiara Valerii

**Affiliations:** 1 Department of Biological, Geological and Environmental Sciences, University of Bologna, Via Selmi 3, 40126 Bologna, Italy; renato.spigarelli@studio.unibo.it (R.S.); mariachiara.valerii2@unibo.it (M.C.V.); 2 Institute for Interdisciplinary Research, Doctoral Programme in Experimental Biology and Biomedicine (PDBEB), University of Coimbra, 3030-789 Coimbra, Portugal; mmagalhaes@cnc.uc.pt; 3 CNC—Center for Neuroscience and Cell Biology, University of Coimbra, 3004-504 Coimbra, Portugal; 4 Coimbra Institute for Clinical and Biomedical Research (iCBR), Clinic Academic Center of Coimbra (CACC), Faculty of Medicine, University of Coimbra, 3000-548 Coimbra, Portugal; celia.cabral@fmed.uc.pt; 5 Center for Innovative Biomedicine and Biotechnology (CIBB), University of Coimbra, 3000-548 Coimbra, Portugal; 6 Centre for Functional Ecology, Department of Life Sciences, University of Coimbra, 3000-456 Coimbra, Portugal; 7 Laboratory of Oncobiology and Hematology, University Clinic of Hematology and Applied Molecular Biology, Faculty of Medicine, University of Coimbra, 3000-548 Coimbra, Portugal; acgoncalves@fmed.uc.pt; 8 iCBR, Group of Environment Genetics and Oncobiology (CIMAGO), Faculty of Medicine, University of Coimbra, 3000-548 Coimbra, Portugal; 9 Microbiology Unit, IRCCS, Azienda Ospedaliero-Universitaria di Bologna, University of Bologna, Via Massarenti 9, 40138 Bologna, Italy; ilariamaria.saracino@studio.unibo.it; 10 Department of Chemical, Pharmaceutical and Agricultural Sciences, University of Ferrara, Via Fossato di Mortara 19, 44121 Ferrara, Italy; bttgdi@unife.it (G.B.); dla@unife.it (A.D.); 11 Department of Life Sciences and Biotechnology, University of Ferrara and LTTA Center, Via Fossato di Mortara 19, 44121 Ferrara, Italy; sarah.beggiato@unife.it

**Keywords:** glioblastoma, eugenol, β-Caryophyllene, essential oils, pharmacokinetics, antitumor activity

## Abstract

This study aimed to investigate β-Caryophyllene (BCA) pharmacokinetics as well as the potential antitumor activity and mechanism of action of BCA and eugenol (EU), alone or in combination, in U87 glioblastoma (GB) cells. The BCA pharmacokinetic was studied by evaluating its concentration profiles in rat blood and cerebrospinal fluid after oral and intravenous administration. EU and BCA antitumor mechanisms were assessed by comparing their effects in U87 GB cells and non-tumoral HMC3 cells. Cell death, cell cycle regulation and mitochondrial membrane potential (MMP) were evaluated using flow cytometry. mRNA levels of target genes were evaluated by qPCR. Secreted cytokines were measured by Luminex^®^. BCA, as well as EU, permeates the brain. EU and BCA affected the viability and proliferation of U87 cells (up to 50%, *p* < 0.001) but not HMC3 cells and showed a synergistic effect. BCA and EU induced G0/G1 cell cycle arrest, increasing apoptosis/necrosis. EU and BCA induced the downregulation of mRNAs encoding for key proteins involved in GB angiogenesis (VEGFA decreased op to 60%, *p* < 0.01), proliferation and progression, and showed anti-inflammatory activity (IL-4 significantly decreased, *p* < 0.001). EU and BCA demonstrated strong and multitarget antitumor activity in U87 cells. Our results provide a strong rationale for the further evaluation of EU and BCA as possible therapeutic molecules in GB management.

## 1. Introduction

Glioblastoma (GB) is the most deadly and frequent primary glioma of the central nervous system (CNS). According to the World Health Organization (WHO), GB is classified as the highest malignancy level (grade 4) glioma [1] and is the most common malignant brain tumor in adults, accounting for approximately 15% of all primary brain tumors [2]. GB is characterized by significant intratumoral heterogeneity, with high variability in genetic, epigenetic and phenotypic profiles. This complexity plays a crucial role in its aggressive behavior and resistance to standard therapies, as tumor subpopulations may exhibit different molecular characteristics that drive adaptive mechanisms and therapeutic evasion. These factors make it challenging to effectively target all malignant cells within the tumor [3].

Currently, the first-line treatment for GB consists of maximal surgical resection followed by cycles of chemoradiotherapy and adjuvant chemotherapy with temozolomide (TMZ) [4]. Despite the advances in treatment, the overall survival for GB patients remains low, with a median survival of 15–21 months after diagnosis. The 5-year survival rate is under 5%, with relapse located within 2 cm of the original surgical cavity in most patients (over 80%). These numbers underline the extent of surgery inefficacy and treatment resistance due to intracerebral localization of this tumor [5]. The therapy’s main limitations are incomplete surgical resection and the development of multidrug resistance (MDR) after TMZ treatment [6]. Furthermore, the toxicity associated with chemotherapy does not allow long-term use for the prevention of relapses. Faced with the impossibility of guaranteeing long-term strategies for the management of GB with pharmacological standard therapy, numerous studies have turned to natural compounds such as curcumin [7], resveratrol [8], quercetin [9], epigallocatechin gallate [10], berberine [11] and 7α-acetoxy-6β-hydroxyroyleanone (Roy) [12]. However, the absorption, distribution, metabolism, excretion and toxicity (ADMET) profiles of natural products are often unpredictable. Poor bioavailability, rapid metabolism, and off-target effects can hinder their clinical development. For this reason, many natural compounds show promising activity in vitro and fail in vivo, mainly due to poor pharmacokinetics or high toxicity [13].

Essential oils (EOs) are mixtures of secondary metabolites produced by aromatic plants, selected after millions of years of evolution as defense mechanisms against environmental threats such as bacteria, fungi, viruses and insect predation. This evolutionary adaptation underlies their diverse biological activities, including antioxidant, anti-inflammatory, immunomodulatory and antitumor properties [14,15]. Despite their high potential, the use of EOs as antitumor agents is hindered by their complex formula and the variability of their composition, which can be influenced by geographical and environmental growth conditions [16]. However, the use of EO-isolated single, active compounds can guarantee experimental reproducibility and a precise evaluation of pharmacokinetics and efficacy [17,18].

Eugenol (4-allyl-2-methoxyphenol) (EU), the major component of clove oil, is a phenylpropanoid that exerts antibacterial, antineoplastic, anti-inflammatory and antioxidant properties, acting as a radical scavenger, apoptosis inducer, voltage-gated sodium channel blocker and NF-kB inhibitor [15]. Moreover, EU also shows a high aptitude to cross the blood–brain barrier (BBB) after intravenous or oral administration [18].

β-Caryophyllene (BCA) is a bicyclic sesquiterpene, present in clove, hemp and black pepper EOs, with demonstrated neuroprotective activities in rotenone-induced Parkinson’s disease (PD) in rats [19]. In this model, BCA neuroprotective effects are exerted by reducing oxidative stress and lipid peroxidation in dopaminergic neurons. BCA has proven to be effective also in Alzheimer’s disease (AD) in vitro models. For example, in PC12 cells overexpressing amyloid-β protein precursor, BCA dramatically increased cell viability by inhibiting the JAK2-STAT3 pathway and enhancing anti-apoptotic signaling, which supports neuronal survival [20]. BCA was also capable of decreasing inflammation in primary microglia cells treated with lipopolysaccharide (LPS) [21] by selectively binding the cannabinoid type 2 receptor (CB2) [22]. 

In the present study, we evaluated the antitumor activity of EU and BCA, alone or in combination, on a standard and well-characterized GB cell line (U87 cells). In order to assess the specificity of these compounds, we also evaluated their effects on a non-tumoral cell line (HMC3). Moreover, a pharmacokinetic study in rats was performed in order to evaluate BCA bioavailability and its ability to penetrate the CNS from the bloodstream.

## 2. Results

### 2.1. Pharmacokinetics of BCA

#### 2.1.1. Intravenous Administration

**Figure 1:**
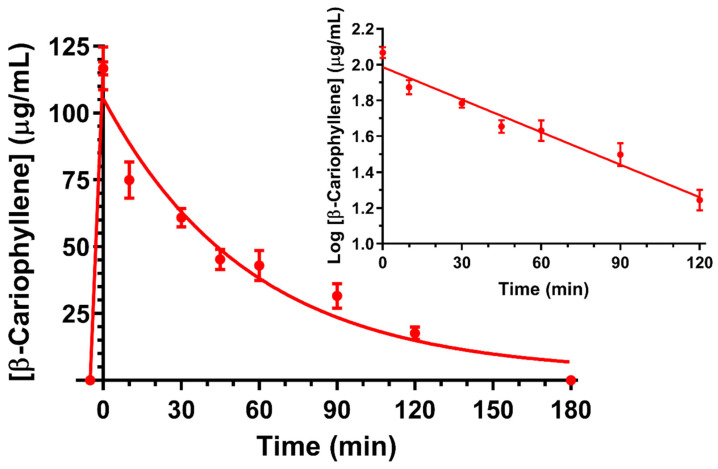
Elimination profile of BCA after 0.4 mg intravenous infusion to rats (2 mg/kg). Data are expressed as the mean ± SE of four independent experiments. The elimination followed apparent first-order kinetics, confirmed by the semilogarithmic plot reported in the inset (n = 7, r = 0.980, *p* < 0.001). The half-life of BCA was calculated to be 49.7 ± 2.0 min.

BCA was administered to rats at a dose of 0.4 mg (2 mg/kg) via intravenous infusion of an ethanolic solution of BCA EO (84% *w*/*w* BCA content), completed within 5 min. At the end of the infusion, a BCA plasmatic concentration of 116.8 ± 5.7 μg/mL was detected. This concentration decreased over time to zero within 180 min (Figure 1) following apparent first-order kinetics, as confirmed by the linearity of the semilogarithmic plot reported in the inset (*n* = 7, *r* = 0.980, *p <* 0.001). The BCA half-life was calculated as 49.7 ± 2.0 min. The AUC value in the bloodstream obtained by the BCA intravenous administration from its infusion starting time to 180 min was 6451 ± 188 μg/mL⋅min (Table 1).

**Figure 2:**
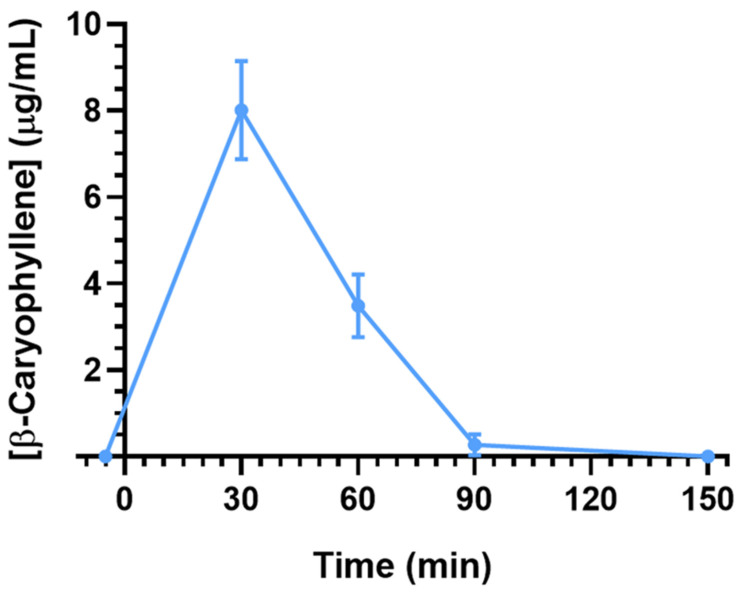
BCA concentrations (μg/mL) detected in the CSF of rats after intravenous administration of a 0.4 mg (2 mg/kg) dose. Data are expressed as the means ± SEM of four independent experiments.

After intravenous administration, BCA was also detected in the CSF. As reported in Figure 2, the BCA concentration increased up to 8.0 ± 0.8 μg/mL (C_max_) within 30 min (T_max_), then decreased to zero within 150 min from the end of infusion. The AUC value of the BCA profile in CSF, from the start of infusion to 150 min, was 377.2 ± 30.5 μg/mL⋅min (Table 1).

The ratio of the BCA concentrations between the CSF and bloodstream (R) at 30 and 60 min from the end of infusion were 0.13 ± 0.01 and 0.08 ± 0.01, respectively. These values, very near to 0.1, highlight the aptitude of BCA to permeate the CNS from the bloodstream following its intravenous infusion to rats.

#### 2.1.2. Oral Administration

BCA was orally administered to male rats as BCA EO (84% *w*/*w* BCA content) dissolved in corn oil, which is known as a suitable vehicle for the dissolution of EO components requiring oral administration [18,23,24]. The oral BCA dose (50 mg/kg; i.e., 10 mg/rat) was chosen taking into account that it is two orders of magnitude below the oral acute LD_50_ value of BCA (estimated over 5000 mg/kg) and also one order of magnitude below the No Observed Adverse Effect Level (NOAEL) dose in chronic administration to rats (700 mg/kg) [25].

**Figure 3:**
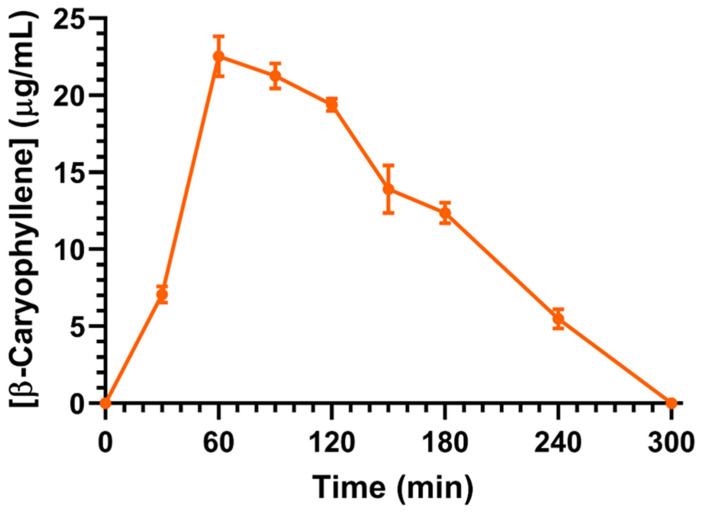
Blood BCA concentrations (μg/mL) within 300 min after oral administration of 10 mg (50 mg/kg) dose to rats. Data are expressed as the means ± SEM of four independent experiments.

The concentration profile of the BCA (μg/mL) over time, obtained in the bloodstream of rats following its oral administration, is reported in Figure 3. The highest concentration in the bloodstream was detected at 60 min (T_max_), with a value of 22.5 ± 0.9 μg/mL (C_max_), which decreased to 0 after 300 min (Figure 3). The AUC value of the BCA profile, here calculated from the time 0 to infinity, was 3410 ± 59 μg/mL⋅min, which allowed the calculation of the absolute bioavailability (F) value of 2.14 ± 0.07% (Table 1).

**Figure 4:**
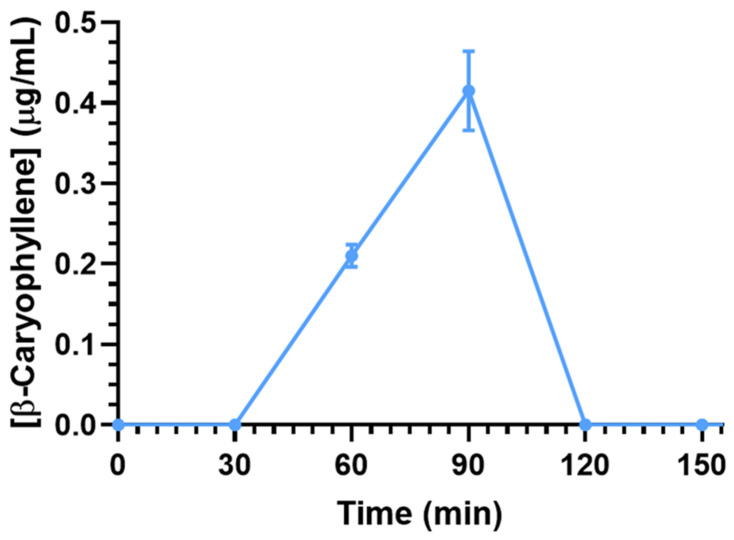
CSF BCA concentrations (μg/mL) within 150 min after oral administration of 10 mg (50 mg/kg) dose to rats. Data are expressed as the means ± SEM of four independent experiments.

Despite the relatively low BCA oral F value, the presence of this compound was quantifiable in the CSF of rats after its oral administration. Indeed, as reported in Figure 4, the C_max_ value of BCA in CSF was 0.42 ± 0.04 μg/mL at 90 min (T_max_) and decreased to zero at 120 min. The AUC value of this profile between 0 and 120 min was calculated as 18.8 ± 1.1 μg/mL⋅min. (Table 1). This value is 0.2% of the normalized AUC value in the CSF obtained from intravenous administration despite an absolute oral bioavailability (F) value of 2.11 ± 0.03%, suggesting that the oral administration reduces BCA’s ability to penetrate the CNS from the bloodstream. This behavior is confirmed by the BCA concentration ratios between CSF and blood (R) at 60 and 90 min of about 0.01 and 0.02, one order of magnitude lower than the R values referred to intravenous administration.

### 2.2. Effect of EU and BCA Co-Administration on the Metabolic Activity of GB Cells

**Figure 5:**
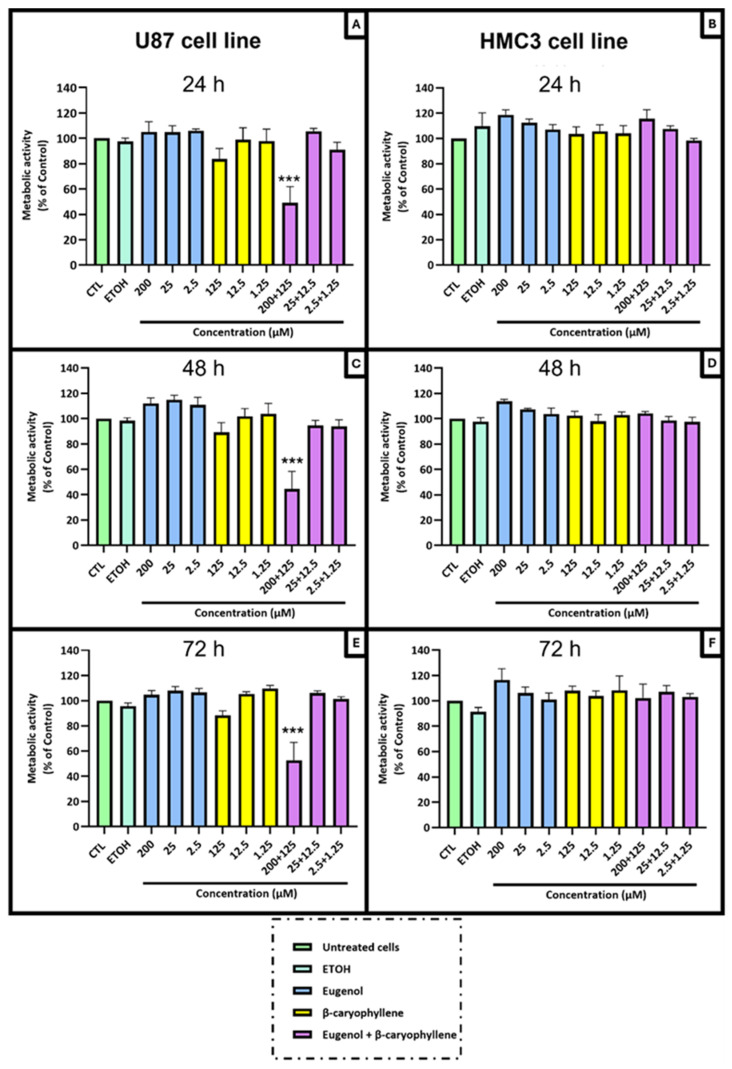
Antiproliferative/cytotoxic activity of EU and BCA, alone or in a combined treatment, in U87 (**A**,**C**,**E**) and HCM3 (**B**,**D**,**F**) cell lines. Cells were treated with EU (2.5, 25 and 200 µM) and BCA (1.25, 12.5 and 125 µM) alone or in combination and subsequently subjected to Alamar blue^®^ assay. Metabolic activity is expressed as a percentage of untreated control cells (CTL). Data are presented as means ± SEM and are representative of at least three independent experiments. *** *p* < 0.001 with respect to the control (CTL) values.

The cytotoxic/antiproliferative potential of EU and BCA, alone and in combination, was evaluated in GB cells (U87 cell line) and non-tumoral microglia cells (HMC3 cell line). No reduction in the metabolic activity with the single compounds was observed at any dose tested (Figure 5A,C,E), while the co-administration of EU and BCA at the higher doses (200 µM and 125 µM, respectively) induced a substantial decrease (up to 60%) in the metabolic activity of the U87 cells (Figure 5A,C,E). This metabolic drop was observed 24 h after cell treatment and was persistent until 72 h. Interestingly, co-administration of EU and BCA did not affect the metabolic activity of HMC3 cells. (Figure 5B,D,F).

**Figure 6:**
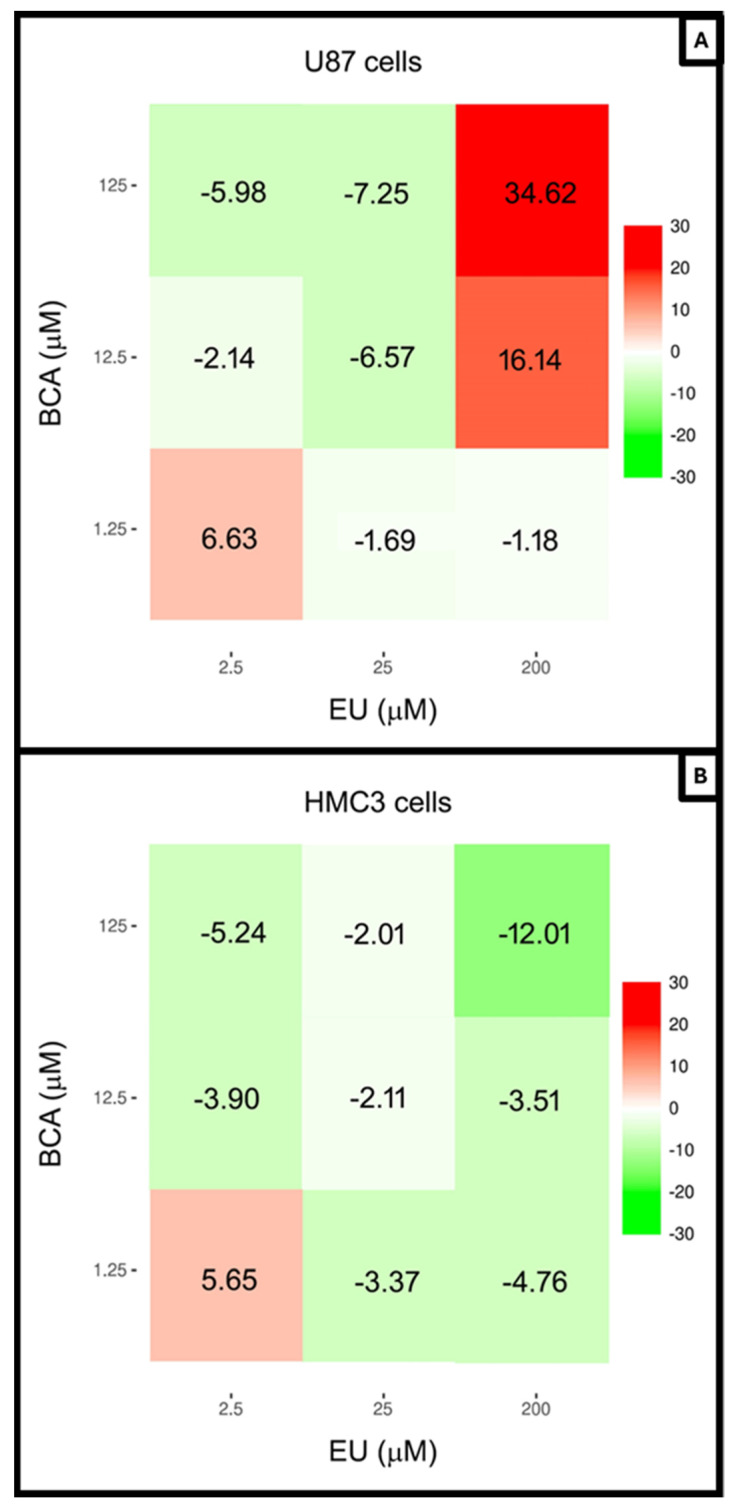
The highest single agent (HSA) synergy scores for EU and BCA were calculated using SynergyFinder software for U87 (**A**) and HMC3 (**B**) cell lines. A synergy score value > 10 is considered synergistic; between −10 and +10 was considered additive, while a synergy score < −10 was considered antagonistic.

The synergy analysis scores obtained with EU and BCA combinations are shown in Figure 6. In the U87 cells, the combined administration of 200 µM EU with both 12.5 and 125 µM BCA resulted in a strong synergism (*p* < 0.001), with the highest score for BCA 125 µM (Figure 6A). No synergies were observed between EU and BCA in non-tumoral cells (Figure 6B), and an antagonistic effect was observed at the maximum concentrations used.

### 2.3. Effects of EU and BCA on Clonogenic Cell Survival Assay

**Figure 7:**
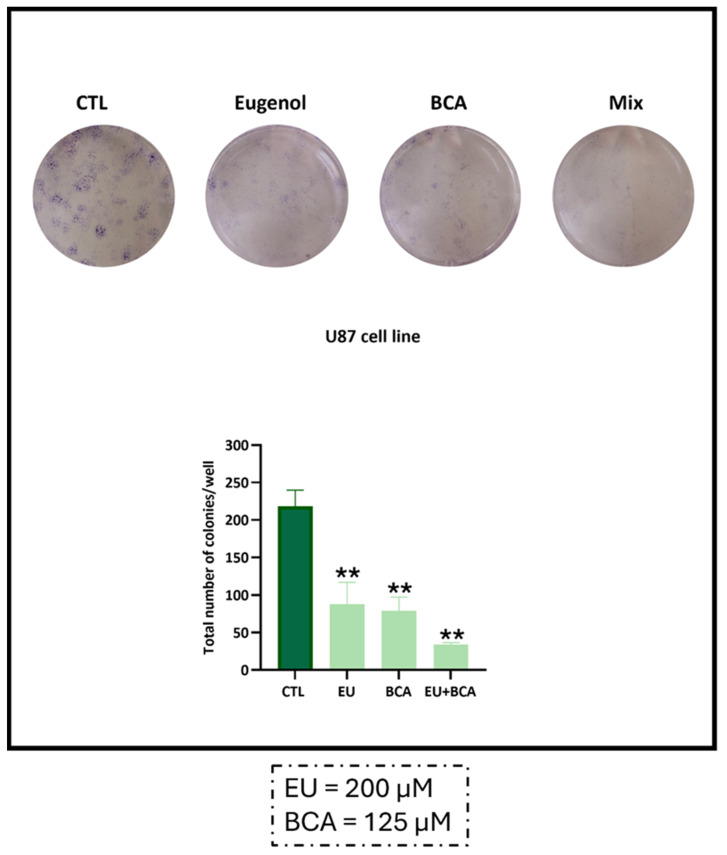
Evaluation of colony formation after treatment with EU and BCA. U87 cells were treated with EU 200 µM and BCA 125 µM, alone or in combination. After a 14-day incubation, colony formation by U87 cells was evaluated by microscopy and the number of colonies was determined using FIJI/ImageJ. ** *p* < 0.01 with respect to the control (CTL) values.

U87 cell incubation with EU and BCA resulted in a strong inhibition of clonal growth, with a decrease of −59.5% (*p* < 0.01) after EU treatment and a decrease of −63.8% (*p* < 0.01) after BCA treatment with respect to the controls. Treatment with EU and BCA in combination reduced colony formation by −84.4% (*p* < 0.01) with respect to the control (Figure 7), supporting a synergistic antiproliferative/cytotoxic activity of these compounds when co-administered.

### 2.4. Effects of EU and BCA on U87 Cell Viability

**Figure 8:**
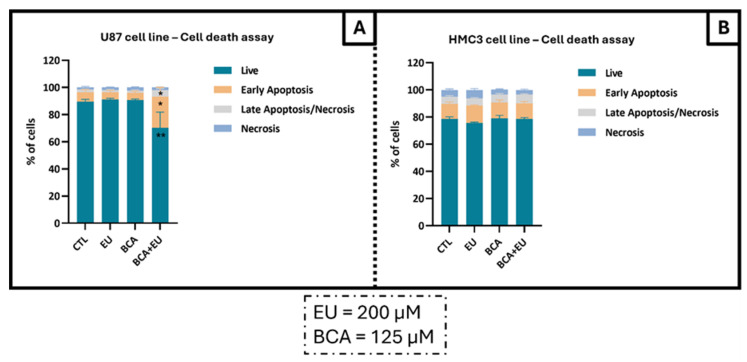
Assessment of cell death profiles in U87 (**A**) and HMC3 (**B**) cell lines treated with EU (200 µM) and/or BCA (125 µM), alone or in combination after 24 h. Cells were co-stained with AV and PI, and the percentage of non-apoptotic cells or apoptotic cells was determined by flow cytometry. * *p* < 0.05 and ** *p* < 0.01 with respect to the control (CTL) values. Data are means ± SEM and are representative of at least three independent experiments.

Treatment with EU (200 µM) or BCA (125 µM) alone did not affect the cell viability of U87 and HMC3 cells. Instead, the co-administration of EU and BCA caused a significant decrease in the percentage of viable cells in the U87 cells after 24 h (Figure 8A). The results show a significant decrease in live cells (−21,6%; *p* < 0.01) and an increase in the percentage of tumor cells in early apoptosis (231.9%; *p* < 0.05) and in late apoptosis (173.2%; *p* < 0.01) (Figure 8A), while the viability of non-tumoral cells was unaffected by the treatments (Figure 8B).

**Figure 9:**
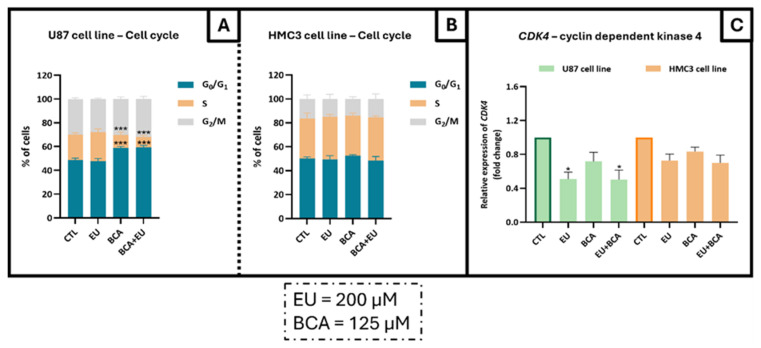
Impact of EU and BCA treatments on cell cycle progression on U87 (**A**) and HCM3 (**B**) cell lines and *CDK4* mRNA levels in both cell types (**C**). Cells were treated with EU (200 µM) and BCA (125 µM) alone or in combination and incubated for 24 h. (**A**,**B**) Cells were stained with PI/RNase and the cell cycle was assessed by flow cytometry. The proportion of cells in the G_0_/G_1_, S and G_2_/M cell cycle phases was expressed as a percentage of the total cell population. (**C**) Relative expression of *CDK4* mRNA levels was assessed by qRT-PCR, and the results were normalized to GAPDH expression. * *p* < 0.05 and *** *p* < 0.001 with respect to the control (CTL) values. Data are means ± SEM and are representative of at least three independent experiments.

Analysis of the U87 cell cycle by using the PI/RNase assay showed no significant effect with EU 200 µM. Treatment with BCA (125 µM) alone or in combination with EU (200 µM) induced cell cycle arrest in the G0/G1 phase in U87 cells, with an increase in the percentage of cells in the G0/G1 phase of 21.1% (*p* < 0.001) and 22.1% (*p* < 0.001), respectively, and subsequently with a decrease in the percentage of cells in the S phase, being −49.4% (*p* < 0.001) with BCA alone and −59.8% (*p* < 0.001) with BCA in combination (Figure 9A). Once again, no effects were observed in the non-tumoral HCM3 cells (Figure 9B).

The qRT-PCR analysis showed that EU, alone or with BCA, induced a substantial downregulation of −49.1% (*p* < 0.05) with EU and −49.9% (*p* < 0.05) with BCA in the *CDK4* mRNA levels in GB cells, while no effects were observed in the BCA-treated cells and in non-tumoral cells (Figure 9C).

### 2.5. Effects of EU and BCA on the Mitochondrial Membrane Potential

**Figure 10:**
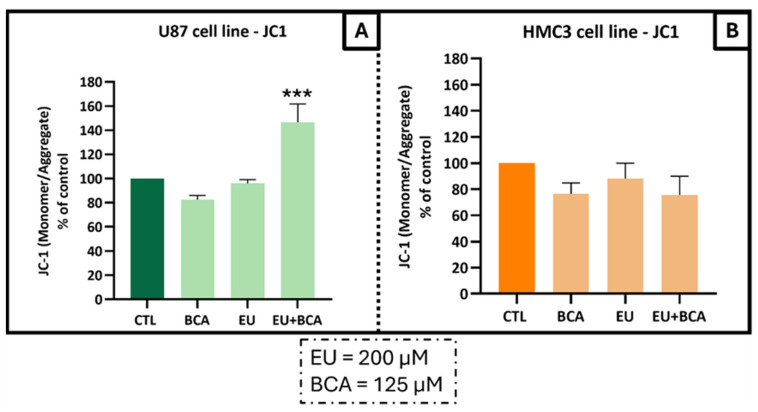
Analysis of the mitochondrial membrane potential in U87 (**A**) and HCM3 (**B**) cell lines. Cells were treated with EU (200 µM) and BCA (125 µM) alone or in combination and incubated for 24 h. Increased values in the monomer/aggregate (M/A) ratio indicate a decrease in the mitochondrial membrane potential. Results are expressed as the M/A ratio of JC-1, which was calculated as the fraction of MFI observed for each molecule. *** *p* < 0.001. The means ± SEM shown are representative of at least three independent experiments.

The evaluation of MMP by flow cytometry with JC-1 dye showed a significant increase (46.5%) in the monomer/aggregate (M/A) ratio, corresponding to a decrease in the MMP and an increase in U87 cells undergoing apoptosis with both EU and BCA. The dissipation of the MMP was followed by an increase in tumor cell apoptosis treatments (Figure 10A); no effect on the MMP in non-tumoral HMC3 cells was observed (Figure 10B).

### 2.6. Effects of EU and BCA on the mRNA Levels on Genes Related to Glioblastoma in U87 Cells

#### 2.6.1. Intrinsic Apoptotic Pathway

**Figure 11:**
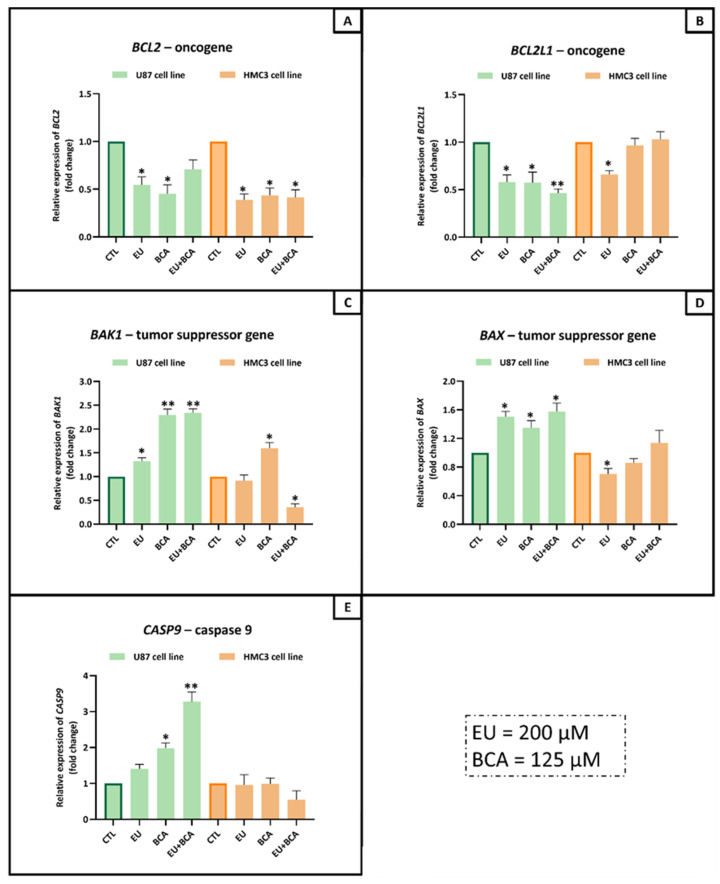
Assessment of *BCL2* (**A**), *BCL2L1* (**B**), *BAK1* (**C**), *BAX* (**D**) and *CASP9* (**E**) mRNA levels in U87 and HMC3 cells treated with EU (200 µM) and BCA (125 µM) for 24 h. Relative expression was assessed by qRT-PCR after normalization with GAPDH expression. * *p* < 0.05; ** *p* < 0.01. Data are the means ± SEM of at least three independent experiments.

In the intrinsic apoptotic pathway (mitochondria-mediated), EU was able to significantly reduce U87-cell mRNA expression of the oncogenes *BCL2* (−45.6%, *p* < 0.05), of *BCL2L1* (−42.2%, *p* < 0.05), and to increase the mRNA expression of the tumor suppressors *BAK1* (+32.3%, *p* < 0.05) and *BAX* (+50.3%, *p* < 0.05); no effect on *CASP9* was observed. The BCA treatment induced a decrease in *BCL2* (−54.8%, *p* < 0.05) and *BCL2L1* (−42.2%, *p* < 0.05) and an increase in *BAK1* (+129.6%, *p* < 0.01), *BAX* (+35.0%, *p* < 0.05) and *CASP9* (+98.5%, *p* < 0.05). The combination of EU and BCA reduced the mRNA expression of *BCL2L1* (−53.6%, *p* < 0.01), *BAK1* (+133.9%, *p* < 0.01), *BAX* (+57.7%, *p* < 0.05) and *CASP9* (227.2%, *p* < 0.01), but no effect on *BCL2* was observed (Figure 11).

In the HMC3 non-tumoral cell line, EU was able to significantly reduce the mRNA expression of *BCL2* (−61.2%, *p* < 0.05), *BCL2L1* (−34.1%, *p* < 0.05) and *BAX* (−29.7%, *p* < 0.05), but no effect on *BAK1* and *CASP9* was observed. The BCA treatment induced a decrease in *BCL2* (−56.2%, *p* < 0.05) and an increase in *BAK1* (+59.8%, *p* < 0.05), but the mRNA expression of *BCL2L1, BAX* and *CASP9* was observed. The combination of EU and BCA reduced the mRNA expression of *BCL2* (−58.7%, *p* < 0.05) and *BAK1* (−64.3%, *p* < 0.05), but no effect on *BCL2L1, BAX* and *CASP9* was observed (Figure 11).

#### 2.6.2. TP53 Pathway

**Figure 12:**
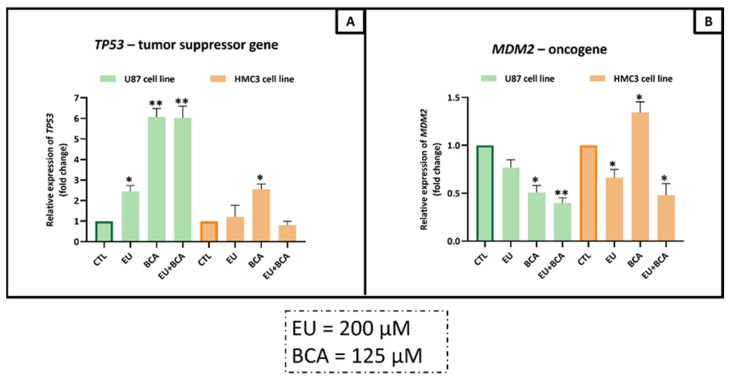
Assessment of *TP53* (**A**) and *MDM2* (**B**) mRNA levels in U87 and HMC3 cells treated with EU (200 µM) and BCA (125 µM) for 24 h. Relative expression was assessed by qRT-PCR after normalization with GAPDH expression. * *p* < 0.05; ** *p* < 0.01. Data are means ± SEM of at least three independent experiments.

In the TP53 pathway, EU was able to significantly increase U87 cell mRNA expression in tumor suppressor gene *TP53* (+143.6%, *p* < 0.05), but no effect on oncogene *MDM2* was observed. The BCA treatment induced an increase in *TP53* (+506.4%, *p* < 0.01) and a decrease in *MDM2* (−49.3%, *p* < 0.05). The combination of EU and BCA increased the mRNA expression of *TP53* (+503.4%, *p* < 0.01) and decreased *MDM2 mRNA expression* (−60.5%, *p* < 0.01) (Figure 12).

In the HMC3 normal cell line, EU significantly reduced the mRNA expression of *MDM2* (−61.2%, *p* < 0.05), but no effect on mRNA expression of *TP53* was observed. The BCA treatment induced an increase in *TP53* (+155.5%, *p* < 0.05) and *MDM2* (+34.2%, *p* < 0.05). The combination of EU and BCA reduced the mRNA expression of *MDM2* (−60.5%, *p* < 0.01), but no effect on *TP53* was observed (Figure 12).

#### 2.6.3. PI3K/AKT/mTOR Pathway

**Figure 13:**
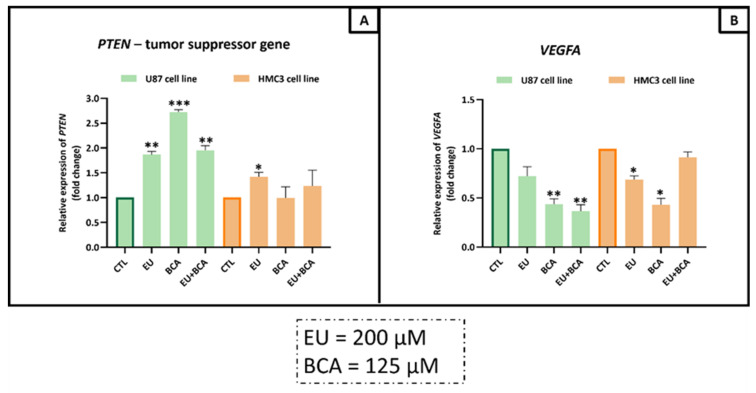
Assessment of *PTEN* (**A**) and *VEGF* (**B**) mRNA levels in U87 and HMC3 cells treated with EU (200 µM) and BCA (125 µM) for 24 h. Relative expression was assessed by qRT-PCR after normalization with GAPDH expression. * *p* < 0.05; ** *p* < 0.01; *** *p* < 0.001. Data are means ± SEM of at least three independent experiments.

In the PI3K/AKT/mTOR pathway, EU induced a significant increase in U87 cell mRNA expression of the tumor suppressor gene *PTEN* (+86.4%, *p* < 0.01), but no effect on *VEGFA* was observed. The BCA treatment induced an increase in *PTEN* (+171.8%, *p* < 0.001) and a decrease in *VEGFA* (−56.5%, *p* < 0.01). The combination of EU and BCA increased the mRNA expression of *TP53* (+95.5%, *p* < 0.01) and decreased *VEGFA mRNA expression* (−63.5%, *p* < 0.01) (Figure 13).

In the HMC3 normal cell line, EU was able to significantly increase the mRNA expression of *PTEN* (+42.2%, *p* < 0.05) and decrease *VEGFA* mRNA expression (−31.5%, *p* < 0.05). The BCA treatment induced a decrease in *VEGFA* (−57%, *p* < 0.05), but no effect on *PTEN* was observed. No effects of combined EU and BCA were detected on the mRNA expression of *TP53* and *VEGFA* (Figure 13).

### 2.7. Cytokines Analysis

**Figure 14:**
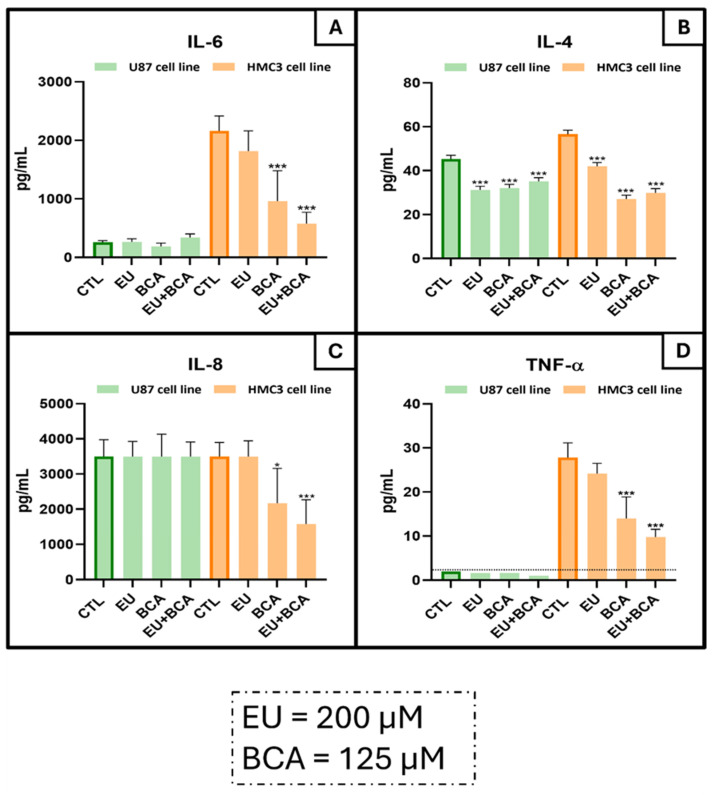
Effects of EU and BCA on the concentration of the inflammatory cytokines IL-6 (**A**), IL-4 (**B**), IL-8 (**C**) and TNF-α (**D**). Cells were treated with EU (200 μM) and BCA (125 μM) alone or in combination and incubated for 24 h. * *p* < 0.05; *** *p* < 0.001 compared to the control condition (untreated cells). Data as means ± SEM are representative of at least three independent experiments.

The effects of EU and BCA, alone and in combination, were also evaluated on cytokine (IL-6, IL-4, IL-8 and TNF-α) secretion from tumoral U87 cells and non-tumoral HMC3 cells. None of the treatments modulated IL-6 secretion in U87 cells, while BCA (−55.5%, *p* < 0.001) and EU + BCA (−73.4%, *p* < 0.001) strongly decreased IL-6 secretion in HCM3 cells (Figure 14A). On the contrary, IL-4 in U87 cells was significantly decreased by all treatments: EU (−31%, *p* < 0.001), BCA (−29.3%, *p* < 0.001) and EU + BCA (−22.5%, *p* < 0.001). Also, in the HMC3 cells, all the tested treatments significantly reduced the IL-4 levels: EU (−26%, *p* < 0.001), BCA (−52.2%, *p* < 0.001) and EU + BCA (−47.3%, *p* < 0.001) (Figure 14B). IL-8 resulted as unaffected by the treatments in U87 cells, while it decreased in HMC3 cells after BCA (−38.0%, *p* < 0.05) and EU + BCA (−55.3%, *p* < 0.001) (Figure 14C). TNF-α secretion in U87 cells remained always below the instrument detection limits (2.26 pg/mL). On the contrary, the HMC3 cells secreted a detectable amount of TNF-α. In these cells, the BCA (−49.7%, *p* < 0.001) and EU + BCA (−64.8%, *p* < 0.001) treatments significantly decreased TNF-α secretion (Figure 14D).

## 3. Discussion

### 3.1. EU and BCA Pharmacokinetics

Numerous efforts have been made to identify compounds for glioblastoma management, but they have been hindered by the inability of many molecules to cross the BBB, limiting the clinical applicability of many potentially effective compounds. In previous studies, we had already demonstrated that EU, one of the compounds investigated in this study, is able to penetrate the CNS from the bloodstream. In this study, pharmacokinetic analyses of BCA revealed that also this compound is able to reach the CNS from the bloodstream. When compared with that of EU [26], the ability of BCA to penetrate the CNS appears reduced, as evidenced by its R values (concentration ratios between CSF and blood) up to one order of magnitude lower than those of EU. 

After its oral administration, only a relatively small amount of BCA (about 2%) was effectively absorbed into the bloodstream of rats from the intestine, and this behavior appears in agreement with the previous hypothesis, based on the poor aqueous solubility, volatility and sensitivity to oxidation of this compound [27,28]. The oral administration of BCA further reduced its ability to penetrate the CNS from the bloodstream, in comparison to the intravenous administration, evidencing a behavior similar to that previously demonstrated for EU [18]. Despite these limitations, the orally administered BCA was, however, detected in the CNS. BCA appears, therefore, a potential candidate for use in CNS pathologies, differently from other main EO components, such as cinnamaldehyde or limonene, whose inability to permeate the CNS after oral administration was evidenced [18]. Considering the pharmacokinetics of EU and BCA and their ability to cross the BBB, their effects on human GB cells were analyzed in this study.

### 3.2. EU and BCA Antitumor Activities

EU and BCA, especially in combination at the concentrations of 200 µM and 125 µM, respectively, exhibited selective cytotoxic and antiproliferative activity on GB cells (U87 cell line), while no cytotoxic effects were observed on microglia cells (HMC3 cell line). In a previous study, *Lavandula pedunculata* (Mill.) Cav. and *Mentha cervine* L. proved effective in inducing apoptosis and cytotoxicity in GB cells, but to date, there are no data on their ability to cross the BBB. Moreover, it is unclear which active compound is responsible for those effects [29]. This can be considered a relevant result since EOs and their single compounds are typically classified as generally recognized as safe (GRAS) by the FDA [30], at least when in vitro. The clonogenic assay showed that both compounds at concentrations of 200 µM (EU) and 125 µM (BCA) reduced the number of clones formed, with particularly strong activity when used in combination. Based on our results, it can be hypothesized that the observed antitumor effect is mediated by a complex set of molecular mechanisms, in line with the well-known multitarget activities of EOs. The cell death analysis conducted on the U87 cells showed that the combination of EU and BCA significantly inhibited cell viability by increasing the cells undergoing apoptosis. The pro-apoptotic activity of the combined treatment with EU and BCA was further supported by a reduction in the MMP. Considering the cytotoxic/antiproliferative effects of the co-administration of EU and BCA in GB cells, we decided to analyze the mechanisms of cell death using the AV/PI assay by flow cytometry. Treatment with BCA alone and in combination with EU also induced the cell cycle arrest of tumor cells in the G0/G1 phase, and the reduction in *CDK4* gene expression is likely to contribute to inhibiting tumor cell growth. *CDK4* plays a key role in promoting the transition from the G1 to S phases, and its overexpression is often linked to uncontrolled proliferation in GB cells [31]. The observed downregulation of *CDK4*, combined with cell cycle arrest, enhances the sensitivity of these cells to targeted treatments [32,33]. When the two compounds were tested alone, the results were conflicting, making it difficult to understand a possible mechanism of action. In fact, when tested alone, EU was able to reduce *CDK4* expression but had no effect on the cell cycle; on the contrary, BCA alone was able to arrest the cell cycle, but no effect was observed on *CDK4* expression. Possible explanations for these observations are that *CDK4’s* reduction was not enough to fall below the threshold necessary to influence the cell cycle [34] and/or that the BCA effect on the cell cycle was mainly exerted by mechanisms not involving *CDK4* inhibition [35,36]. Since EU and BCA induced selective cell death by apoptosis in GB cells via the mitochondria-dependent pathway, we evaluated their effects on the specific targets involved in tumorigenesis, such as the tumor suppressor genes and oncogenes involved in the activation of apoptotic mechanisms. The genes analyzed, *BCL2*, *BCL2L1*, *BAK1*, *BAX*, *PTEN*, *TP53*, *MDM2*, *CASP9* and *VEGFA*, play key roles in fundamental processes for the survival and proliferation of GB tumor cells and are involved in three different pathways: the intrinsic apoptotic pathway (mitochondrial pathway), the TP53 pathway and the PI3K/AKT/mTOR pathway, all contributing to tumor growth, drug resistance and the ability of the tumor to evade programmed cell death [37,38,39,40,41].

### 3.3. Activated Apoptotic Pathway

The intrinsic apoptotic pathway (mitochondria-mediated) is crucial for regulating programmed cell death (apoptosis), which is often dysfunctional in GB. *BCL2*, *BCL2L1*, *BAK1*, *BAX* and *CASP9* are all genes involved in this pathway and have different mechanisms of action [42]. Anti-apoptotic genes, such as *BCL2* and *BCL2L1*, are frequently overexpressed in GB, preventing the activation of apoptosis even in the presence of significant cellular damage. As a result, tumor cells can survive and proliferate even when treated with drugs that would normally induce cell death. In our study, both *BCL2* and *BCL2L1* mRNA levels were reduced following treatments with EU and BCA [43,44]. In the same pathway, tumor suppressor genes encoding for pro-apoptotic proteins such as *BAX* and *BAK1* are inactivated in GB cells, contributing to resistance to radiotherapy and chemotherapy [42,45,46]. Our results showed a significant increase in *BAX* and *BAK1* mRNA following the tested treatments, as well as of *CASP9,* which is involved in apoptosis as an initiator caspase [42,47]. These results suggest that these compounds are active on different targets of this specific pathway. The *TP53* pathway regulates cell cycle arrest and apoptosis and is activated following DNA damage or cellular stress [48,49]. This pathway is regulated by *MDM2,* which physiologically degrades p53. *TP53* is often downregulated in GB cells due to mutations or overexpression of *MDM2*; this leads to the cell’s inability to initiate apoptosis, thus contributing to the accumulation of mutations and tumor progression [48]. Moreover, the downregulation of *TP53* contributes to tumor cell resistance to those therapies that induce apoptosis through DNA damage [50]. Following treatments with EU and BCA alone and in combination, *TP53* mRNA expression strongly increased, while *MDM2* mRNA levels were decreased. The PI3K/AKT/mTOR pathway plays a central role in regulating cell growth and protein synthesis by stimulating mTOR. This pathway is involved in cell cycle progression, supporting the production of proteins required for growth, metabolism and replication; moreover, it has a critical role in promoting cell survival and inhibiting apoptosis [51,52,53]. This pathway can be overactivated in GB due to the downregulation of *PTEN*, which physiologically acts as a tumor suppressor by inhibiting the PI3K/AKT/mTOR pathway [54]. As a consequence, cells acquire resistance to apoptotic stress and become able to evade growth regulation mechanisms. Overall, this contributes to drug resistance [50,55]. Our results showed a significant increase in *PTEN* gene expression in cells treated with EU and BCA alone or in combination.

### 3.4. Anti-Angiogenic Effects

Since GB tumors are also characterized by a high VEGF secretion profile [56], we explored the impact of EU and BCA, alone or in combination, on the expression of *VEGFA* mRNA levels, a gene upregulated in GB cells and involved in tumor growth and vascularization [57]. The significant reduction in *VEGFA* observed after treatment with BCA alone and in combination is relevant since this gene is involved in the PI3K/AKT/mTOR pathway as well as in the angiogenesis pathway. This gene is crucial for tumor growth and is one of the main targets of anti-angiogenic therapies [41,58].

### 3.5. Anti-Inflammatory Effects

The cytokine analysis revealed a high basal secretion of IL-4, IL-6, IL-8, and TNF-α by HMC3 microglial cells but not by U87 GB cells, in which only IL-4 and IL-8 secretion was relevant. When treated with EU and BCA, both individually and in combination, we noticed decreases in all cytokine levels in the HMC3 cells. However, in the U87 cells, of the two elevated cytokines, only the IL-4 levels decreased with treatment. These observations at baseline may be explained by the intrinsic characteristics of the two cell types: in fact, microglial cells like HMC3 play a crucial role in immune surveillance within the CNS. Even without external stimuli, they can produce cytokines such as IL-6, IL-8 and TNF-α at basal levels to maintain neuroprotective and immunoregulatory functions [59,60]. On the other hand, GB cells often develop mechanisms to evade the immune system and tend to suppress pro-inflammatory cytokines like IL-6 and TNF-α to avoid triggering an immune response that could lead to their elimination [61]. However, the high secretion of IL-8 also after the treatments in U87 cells could be explained by the role of this cytokine in promoting angiogenesis and tumor progression. In fact, IL-8 stimulates the proliferation and migration of endothelial cells, contributing to the formation of new blood vessels that supply the tumor with nutrients and oxygen [62]. Elevated levels of IL-4 in both HMC3 and U87 cells possibly reflect its complex role in cellular biology. In non-tumoral cells, IL-4 has a pivotal role in modulating the immune response [63]. In GB, an increase in IL-4 contributes to tumor growth and immune suppression. In fact, it promotes the polarization of macrophages toward the M2 phenotype, which supports an anti-inflammatory environment conducive to tumor progression [64]. IL-4 activates several signaling pathways in GB cells, including the JAK/STAT6 pathway, whose activation leads to the transcription of genes that promote cell survival and proliferation; the PI3K/AKT pathway, which facilitates cell growth and resistance to apoptosis; and the MAPK/ERK pathway, which promotes cell division and differentiation [64,65]. These pathways collectively enhance GB cell survival, proliferation and resistance to therapies. The differential response to treatment between HMC3 and U87 cells suggests that EU and BCA may modulate specific signaling pathways differently in these cell types. In HMC3 cells, the reduction in all cytokine levels indicates a broad compound-induced anti-inflammatory effect, potentially suppressing microglial activation. In U87 cells, the selective decrease in IL-4, but not IL-8 upon treatment implies that EU and BCA may specifically interfere with the apoptotic signaling pathways, which could reduce tumor growth.

### 3.6. Limitations to the Study

This is an in vitro study on tumor cells in culture. As in all studies of this type, the complexity of the organ is lost, as are the complex relationships between the tumor and the extracellular matrix. Nevertheless, the two cell types (GB and control) were carefully chosen to have a similar proliferation rate, and therefore, the results obtained demonstrate selective antitumor activities of EU and BCA at the concentrations tested. Although both compounds cross the blood–brain barrier, in vivo-specific delivery systems will probably be needed to obtain similar concentrations inside the brain, especially for BCA, which has greater difficulty than EU in reaching the brain.

## 4. Materials and Methods

### 4.1. Materials

**Figure 15:**
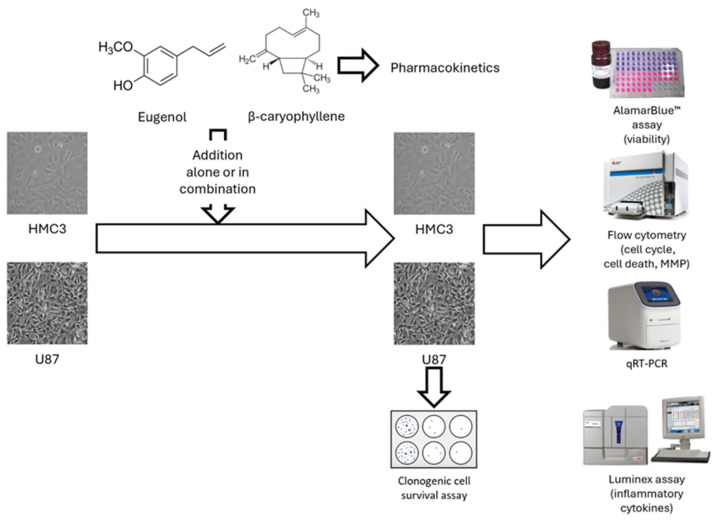
General scheme of the experimental design of the in vitro study. Eugenol and β-Caryophyllene were added to HMC3 (normal) and U87 (glioblastoma) cells, alone or in combination for 24–72 h. After treatment, cell viability, cell cycle analysis, real-time qPCR on different genes involved in tumor progression/angiogenesis, mitochondrial membrane potential (MMP) and cytokines secretion were analyzed.

EU and BCA were provided by Xeda International S.A. (Saint-Andiol, France). EU pure (98% *w*/*w*, density = 1.06 g/mL) and BCA pure (98% *w*/*w*, density = 0.902 g/mL, MW = 204.35 g/mol) were stored at 4 °C and protected from light to maintain stability and prevent oxidation. HPLC-grade acetonitrile (CH_3_CN) was obtained from Carlo Erba Reagents S.A.S. (Milan, Italy). The Sartorius Arium^®^ Advance EDI system (Sartorius Lab Instruments GmbH & Co, KG, Göttingen, Germany) was used to obtain ultrapure water (H_2_O) for the HPLC analysis. All other reagents were of analytical grade from Merck Life Sciences Srl (Milan, Italy). A scheme of the study’s experimental design is shown in Figure 15.

### 4.2. BCA Pharmacokinetic in Rats

Male Sprague-Dawley rats (200–250 g) were purchased from the Charles River laboratories (Calco, Italy). BCA was administered to rats by intravenous and oral routes in order to evaluate the pharmacokinetic profiles in the bloodstream and cerebrospinal fluid (CSF). For the intravenous administration, BCA EO (84% *w*/*w* BCA content) was dissolved in ethanol to obtain a final BCA concentration of 8 mg/mL. A group (n = 4) of rats, fasted for 24 h, was anesthetized during the experimental period and then received via a femoral intravenous infusion (rate = 10 μL/min; 5 min) 50 µL of the 8 mg/mL ethanolic solution (BCA dose 2 mg/kg). This dosage was chosen basing on a previous study where BCA was intravenously administered to rats at a dose of about 1.5 mg/kg as a minor component of *Artemisiae argyi Folium* EO (5.3% *w*/*w*) [66]. For oral administration, BCA EO was dissolved in corn oil to obtain a final BCA concentration of 20 mg/mL. A total of 500 µL of this corn oil solution was orally administered by gavage to a group (n = 4) of rats kept fasting for 24 h (BCA dose 50 mg/kg). 

At the end of the intravenous and oral administrations and at fixed time points, blood samples (100 μL) were collected. Moreover, CFS samples (about 30 μL) were withdrawn using the cisternal puncture method previously described [67]. This method requires a single needle stick and allows the collection of serial (about 30 μL) CSF samples that are virtually blood-free [68]. A total volume of a maximum of 120 μL of CSF/rat (i.e., four 30 μL samples/rat) was collected during the experimental session, choosing the time points (n = 4) to allow the restoration of the CSF physiological volume. The CSF samples (10 μL) were immediately analyzed via HPLC (see below) for the quantification of BCA. For the BCA analysis in the bloodstream, the blood samples (n = 4) were immediately added to 200 μL of ice-cold CH_3_CN, then 100 μL of the internal standard dissolved in CH_3_CN (100 μM GER-UDCA, obtained by the conjugation of geraniol with ursodeoxycholic acid) was further inserted [69]. The samples were centrifuged at 16,000× *g* for 5 min, then about 300 μL of the supernatant was withdrawn and further centrifuged. Finally, 10 μL was analyzed via HPLC (see below) for BCA quantification. A blood sample (100 μL) was collected from each rat before the administration of the compound and was used as a control. The control samples were immediately added to 300 μL of ice-cold CH_3_CN in the absence of the internal standard and then treated as described above.

The BCA was quantified by the HPLC method, using a chromatographic apparatus that consisted of a modular system (model LC-10 AD VD pump and model SPD-10A VP variable wavelength UV−vis detector; Shimadzu, Kyoto, Japan) and an injection valve with a 20 μL sample loop (model 7725; Rheodyne, IDEX, Torrance, CA, USA). The separations were performed on a 5 μm Hypersil BDS C-18 column (150 mm × 4.6 mm i.d.; ThermoFisher Scientific S.p.A., Milan, Italy) equipped with a guard column packed with the same Hypersil material at room temperature by injecting 10 µL of the samples into the apparatus. Data were acquired and processed using CLASS-VP software, version 7.2.1 (Shimadzu Italia, Milan, Italy), installed on a personal computer. The mobile phase consisted of an isocratic mixture of water and acetonitrile at a ratio of 10:90 (*v*/*v*), with a flow rate of 1 mL/min. The detector was set at 205 nm. The retention times obtained were 7.5 min for BCA and 5.6 min for GER-UDCA. 

The chromatographic precision was evaluated by repeated analyses (n = 6) of the same sample solution containing 100 μM (20.435 µg/mL) BCA dissolved in a mixture of water and CH_3_CN 25:75 (*v*/*v*). The chromatographic precision, expressed as the relative standard deviation (RSD) value, was 0.84%. The calibration curve of the peak areas versus concentration was generated in the range from 0.1 to 600 μM (0.020 μg/mL–122.6 μg/mL) for the BCA dissolved in a mixture of water and CH_3_CN 25:75 (*v*/*v*) and resulted linear (n = 9, r = 0.998, *p* < 0.0001). 

A preliminary analysis performed on blank CSF and blood samples showed that their components did not interfere with the retention times of the BCA and its internal standard (GER-UDCA). For the CSF simulation, standard aliquots of balanced solution (Dulbecco’s phosphate-buffered saline-DPBS-without calcium and magnesium) in the presence of 0.45 mg/mL BSA were used [70,71]. The calibration curve of the peak areas versus concentration in CSF simulation fluid of the BCA was generated in the range of 0.5 to 50 μM (0.10 to 10.2 μg/mL) and resulted linear (n = 7, r = 0.996, *p* < 0.0001). Recovery experiments from blood samples were performed, comparing the peak areas extracted from the blood test samples with those obtained by injection of equivalent concentrations of BCA dissolved in a water–CH_3_CN mixture (25:75 *v*/*v*). The average recovery of BCA ± SD, obtained by five different BCA concentrations ranging from 10 µM and 300 µM (2.044 μg/mL–61.305 μg/mL), was 76.16 ± 5.71%. Therefore, the BCA blood concentrations were referred to as the peak area ratio with respect to its internal standard (GER-UDCA). The calibration curve was constructed by using eight different concentrations of BCA in whole blood at 4 °C, ranging from 1 to 600 μM (0.204 to 122.6 μg/mL), and resulted linear (n = 8, r = 0.994, *p* < 0.001).

The nonlinear regression (exponential decay) of the concentration values in appropriate time ranges after intravenous infusion was used to calculate the in vivo half-life (t_1/2_) of BCA in the bloodstream of rats and was then confirmed by linear regression of the log concentration values *versus* time (semilogarithmic plot). The area under concentration curves (AUC, μg/mL∙min) related to intravenous and oral administrations of BCA in the bloodstream or CSF of rats were calculated by the trapezoidal method. The absolute bioavailability value (F) referred to BCA following the oral administrationwas obtained as the ratio between the oral AUC and intravenous AUC values obtained for the compound in the bloodstream, normalized with respect to the doses, according to the following Equation [72]:

$$F=\frac{{AUC}_{oral}}{{AUC}_{IV}}\cdot \frac{{dose}_{IV}}{{dose}_{oral}}$$

This study was conducted in accordance with the Declaration of Helsinki and was carried out in accordance with the current Italian legislation (D.L. 26/2014) that allows experimentation on laboratory animals only after approval by the Ministry of Health (Rome, Italy; protocol n: 793/2018-PR), and is in strict accordance with the European Council Directives (n. 2010/63/EU) on animal use in research. 

All efforts have been made to reduce the number of animals and their suffering.

### 4.3. In Vitro Evaluation of EU and BCA Effects on GB and HMC3 Cells

#### 4.3.1. Cell Cultures

The U87 and HMC3 cell lines were purchased from the American Type Culture Collection (ATCC, Manassas, VA, USA). The cells were cultivated in Dulbecco’s Modified Eagle’s Medium-high glucose (DMEM-HG) (Biowest, Nuaillé, France), supplemented with 10% (*v*/*v*) of heat-inactivated fetal bovine serum (FBS) (Biowest, Nuaillé, France) and 1% (*v*/*v*) of penicillin–streptomycin (Sigma, St. Louis, MO, USA), and maintained in a humidified incubator at 37 °C and 5% CO_2_. All experiments were performed in cultures in log-phase growth. The U87 and HMC3 cell lines have a similar mean doubling time of about 30 h, as previously reported [59,73].

#### 4.3.2. Metabolic Activity Assay

To evaluate the cytotoxic/antiproliferative potential of BCA and/or EU, the U87 and HMC3 cells were seeded, in triplicate, into 48-well culture plates (4 × 10^4^ cells/well). After 24 h, the medium was replaced, and the cells were treated either with the vehicle (ethanol) or with different concentrations of EO single molecules, diluted in ethanol alone (EU: 2 µM, 20 µM, 200 µM; BCA: 1.25 µM, µM, 125 µM) or in combination (EU 2 µM and BCA 1.25 µM; EU 20 µM and BCA 12.5 µM; and EU 200 µM and BCA 125 µM) in order to evaluate the possible synergistic effects. The final concentration of ethanol in the cell medium was kept lower than 0.1%. Non-treated cells were considered the control condition. 

Metabolic activity was evaluated using a modified Alamar blue^®^ assay, as previously described [74]. Briefly, a solution of DMEM-HG with 10% (*v*/*v*) of resazurin salt dye stock solution (Sigma, St. Louis, MO, USA) (0.1 mg/mL) was prepared and added to each well after 24 h, 48 h and 72 h treatments. After 2 h of incubation at 5% CO_2_ and 37 °C, the absorbance was read at 570 and 600 nm in a BioTeck (BioTek Instruments, Inc., Winooski, VT, USA). The absorbance results were obtained using the Gen5 program. The metabolic activity was then calculated by the following equation:

$$Metabolicactivity\;\left(\%\right)=\frac{\left(A570-A600\right)\;oftreated\;cells}{\left(A570-A600\right)\;ofcontrol\;cells}\times 100$$

#### 4.3.3. Clonogenic Cell Survival Assay

An evaluation of the antiproliferative effect via clonogenic assay on the tumoral U87 cells was performed using an adapted protocol from the previously described procedure [75]. Briefly, U87 cells (0.8 × 10^3^ cells/well) were seeded into 6-well plates and incubated for 24 h. After the incubation, based on the results obtained from the cell viability assay and synergistic score, the cells were treated either with EO single compounds diluted in ethanol alone (EU 200 µM and BCA 125 µM) or in combination for 14 days with the media being changed every 2/3 days. The cells without treatment were considered the control condition. Following the incubation, the cells were washed with PBS, fixed with paraformaldehyde (PFA) and stained with 0.1% crystal violet solution. The number of colonies was counted by microscopy, and the surviving fraction (SF) was calculated as the mean colonies/number of cells seeded.

#### 4.3.4. Cell Cycle Analysis

Analysis of the effect of BCA and/or EU on the cell cycle was assessed with flow cytometry using propidium iodide (PI)/RNase solution, according to the manufacturer’s protocol (Immunostep, Salamanca, Spain), as previously described [12]. HMC3 and U87 cells (30 × 10^4^ cells/well) were seeded in triplicate into 6-well plates and incubated for 24 h at 37 °C and 5% CO_2_. Thereafter, the cells were treated either with EU (200 µM) or BCA (125 µM) alone or in combination (EU 200 µM + BCA 125 µM). After 24 h, the cells were detached and fixed in 70% ethanol for 60 min at 4 °C, washed twice with PBS, and then stained with 500 µL of PI/RNase solution. The results were acquired using CellQuest software and expressed as the calculated percentage of the cell population in each cell cycle phase (G_0_/G_1_, S and G_2_/M) according to the PI intensity.

#### 4.3.5. Cell Death Assay

The Annexin V(AV)/PI assay with flow cytometry [76] was used to investigate the potential mechanism underlying the BCA- and/or EU-induced cell death. This method enables the separation of living cells from cells in apoptosis or necrosis based on the integrity and permeability of their plasma membrane [77]. HMC3 and U87 cells were seeded in duplicate into 12-well plates (10 × 10^4^ cells/well). After 24 h of incubation at 37 °C and 5% CO_2_, the medium was replaced, and the cells were treated either with EU (200 µM) or BCA (125 µM), alone and in combination (EU 200 µM + BCA 125 µM). Following the 24 h treatment, the cells were co-stained with AV-APC and PI according to the manufacturer’s protocol (Biolegend, San Diego, CA, USA). Briefly, the cells were resuspended in binding buffer (100 µL) and incubated with AV-APC solution (5 µL) and PI solution (2 µL) for 15 min at room temperature. Then, the cells were diluted in a binding buffer (400 µL). A six-parameter, four-color FACSCalibur flow cytometer (Becton Dickinson, San Jose, CA, USA) was used, and at least 10,000 events were collected by acquisition using CellQuest software (Becton Dickinson, San Jose, CA, USA). The results were analyzed with the Paint-a-Gate software and expressed in percentage (%).

#### 4.3.6. Mitochondrial Membrane Potential Evaluation

The following protocol aimed to investigate the impact of EO compounds on the mitochondrial membrane potential (MMP). The MMP (ψmit) was evaluated in HMC3 and U87 cells using 5,5,6,6′-tetrachloro-1,1′,3,3′ tetraethylbenzimi-dazoylcarbocyanine iodide (JC-1) (Molecular Probes), as previously described by Gonçalves and collaborators [78]. JC-1 is a cationic and lipophilic dye that, in healthy cells, accumulates and forms aggregates within the mitochondria, while, in apoptotic cells, it cannot accumulate inside the mitochondria due to the loss of the MMP, thus maintaining its monomeric form in the cytosol [77]. Cells were seeded in triplicate into 6-well plates (30 × 10^4^ cells/well) and incubated for 24 h. Then, the medium was discarded, and the cells were treated either with EU (200 µM) or BCA (125 µM) alone and in combination (EU 200 µM + BCA 125 µM). After 24 h of incubation, the cells were washed twice with PBS, centrifuged at 3,450 rpm for 5 min and incubated with JC-1 at a final concentration of 5 μg/mL for 15 min at room temperature in the dark. The cells were then washed twice with PBS, centrifuged and resuspended in a total volume of 500 μL of PBS and analyzed by flow cytometry.

#### 4.3.7. Quantitative Real-Time PCR

An analysis of the genes involved in angiogenesis, tumor progression and proliferation were performed. In particular, we analyzed the genes involved in the cell cycle and in three different apoptotic pathways: *B-cell lymphoma-2* (*BCL2*), *Bcl-2-like protein* 1 (*BCL2L1*), *Bcl-2 Associated X-protein* (*BAX*), *BCL2 Antagonist/Killer 1* (*BAK-1*) and *Caspase 9* (*CASP9*) as the key genes of the intrinsic mitochondrial pathway; and *Phosphatase and tensin homolog* (*PTEN*), *Tumor protein p53* (*TP53*), *Murine double minute 2* (*MDM2*), *Cyclin-dependent kinase 4* (*CDK4*) and *Vascular endothelial growth factor A* (*VEGFA*) mRNA levels were assessed by quantitative real-time PCR (qRT-PCR). HMC3 and U87 cells were seeded with a density of 1 × 10^6^ cells per well and incubated for 24 h at 37 °C and 5% CO_2_. Thereafter, the cells were treated either with EU (200 µM) or BCA (125 µM) alone and in combination (EU 200 µM + BCA 125 µM). Total RNA was extracted using the TripleXtractor solution (GRISP, Lisbon, Portugal) according to the manufacturer’s protocol. RNA was converted into cDNA through the Xpert cDNA Synthesis Supermix (GRISP, Lisbon, Portugal). A total of 100 ng of cDNA was amplified by qRT-PCR using the primers reported in Table 2. Each optimized reaction was performed using Xpert Fast SYBR Green Mastermix 2X with ROX (GRISP, Lisbon, Portugal) and subjected to the amplification protocol described by the manufacturer, using a melting temperature of 60 °C. The relative gene expression was determined by the 2^−ΔΔCt^ method and normalized on *Glyceraldehyde-3-Phosphate Dehydrogenase* (*GAPDH*), which was the endogenous reference and relative to the untreated cells (control condition).

#### 4.3.8. Determination of Inflammatory Cytokines on Conditioned Media

In order to determine the impact of BCA and/or EU on inflammatory cytokines levels, cytokine expression analyses (L-6, IL-4, IL-8 and TNF-α) were performed using a customized detection panel purchased from Bio-techne (Minneapolis, MN, USA).

The cells were seeded, in triplicate, into 24-well culture plates (8 × 10^4^ cells/well). After 24 h, the medium was replaced and the cells were treated either with the vehicle (ethanol) or with different concentrations of EO single compounds alone (EU: 2 µM, 20 µM, 200 µM; and BCA: 1.25 µM, 12.5 µM, 125 µM) or in combination (EU 2 µM + BCA 1.25 µM; EU 20 µM + BCA 12.5 µM; and EU 200 µM + BCA 125 µM). The final concentration of ethanol in the cell medium was kept lower than 0.1%. The ethanol 0.1%-treated cells were considered the control condition.

After the 24 h treatment, supernatants were collected and centrifuged at 4,000× *g* for 10 min, and the samples were analyzed with the BioPlex 200 instrument (BioRad^®^, Hercules, CA, USA) following the manufacturer’s protocol.

#### 4.3.9. Statistical Analysis

All data were analyzed using the software GraphPad Prism v.9.00 (GraphPad Software, San Diego, CA, USA). All in vitro experiments were performed in triplicate, and the acquired results were expressed as means ± SEM. The EO combination responses were calculated based on the highest single agent (HSA) model using SynergyFinder [79]. A HSA synergy score value of > 10 was considered synergistic; between −10 and +10 was considered additive, and a synergy score < −10 was considered antagonistic. The statistical analysis was performed using a Student’s t-test and one-way and two-way ANOVAs, using the unpaired comparison and the multiple comparisons tests Tukey and Dunnett, respectively. A value of *p* < 0.05 was considered significant.

## 5. Conclusions

Overall, we have demonstrated the antitumor potential of EU and BCA on an in vitro model of GB. EU and BCA have proven to be molecules with multitarget antitumor and anti-inflammatory activity. In this context, it is particularly relevant that the association of EU and BCA has shown interesting synergistic effects. The low toxicity in vitro and in vivo models—together with their ability to cross the BBB—makes these compounds attractive and provides a strong rationale for their evaluation in animal models. In particular, future research on EU and BCA synergy could follow two different paths. The first one should be based on the use of animal models of GB to test the antitumor activities of EU + BCA, alone or in association with other natural or pharmacological therapeutic molecules. The second one, rather, should adopt inflammation-based experimental models of neurodegeneration in rodents. EU + BCA shows compelling anti-inflammatory effects on microglial cells and could, therefore, also have beneficial effects in counteracting neurodegeneration.

## Figures and Tables

**Table 1:** Pharmacokinetic parameters referred to the bloodstream or cerebrospinal fluid (CSF) of rats after intravenous or oral administration of BCA as essential oil. Data are reported as the mean ± SE of four independent experiments. C_0_: concentration at the end of infusion; C_max_: maximum concentration obtained in the bloodstream or in the CSF; t_1/2_: half-life; T_max_: time of C_max_; AUC: area under concentration; F: absolute bioavailability; R: ratio of concentration between CSF and bloodstream at CSF T_max_.

	Intravenous administration	Oral administration
Dose (mg/kg)	2	50
Bloodstream		
C_0_ (µg/mL)	116.7 ± 1.7	-
C_max_ (µg/mL)	-	22.53 ± 0.09
t_1/2_ (min)	49.7 ± 2.0	-
T_max_ (min)	-	60
AUC (µg/mL∙min)	6451 ± 188	3410 ± 59
F (%)	-	2.14 ± 0.07
Cerebrospinal fluid (CSF)		
C_max_ (µg/mL)	8.0 ± 0.8	0.42 ± 0.04
T_max_ (min)	30	90
AUC (µg/mL∙min)	377.2 ± 30.5	18.8 ± 1.1
R	0.13 ± 0.01	0.019 ± 0.002

**Table 2:** Sequence of the primers used in quantitative real-time PCR (qRT-PCR).

Gene	Forward (5′-3′)	Reverse (5′-3′)
CDK4	AGCCGAAACGATCAAGGAT	GCTTGACTGTTCCACCACTTG
BCL2	GAGGATTGTGGCCTTCTTTGAG	AGCCTCCGTTATCCTGGATC
BCL2L1	GCCACTTACCTGAATGACCACC	AACCAGCGGTTGAAGCGTTCCT
BAK1	TTACCGCCATCAGCAGGAACAG	GGAACTCTGAGTCATAGCGTCG
BAX	TCAGGATGCGTCCACCAAGAAG	TGTGTCCACGGCGGCAATCATC
PTEN	TGAGTTCCCTCAGCCGTTACCT	GAGGTTTCCTCTGGTCCTGGTA
TP53	CAGCACATGACGGAGGTTGT	TCATCCAAATACTCCACACGC
MDM2	TGTTTGGCGTGCCAAGCTTCTC	CACAGATGTACCTGAGTCCGATG
CASP9	GTTTGAGGACCTTCGACCAGCT	CAACGTACCAGGAGCCACTCTT
VEGFA	TGCAGATTATGCGGATCAAACC	TGCATTCACATTTGTTGTGCTGTAG
GAPDH	GTCTCCTCTGACTTCAACAGCG	ACCACCCTGTTGCTGTAGCCAA

## Data Availability

The datasets generated during the current study are available from the corresponding author upon reasonable request.
